
JOURNAL INFORMATION
==============================
Journal ID: Wirtschaftsdienst

Wirtschaftsdienst

EISSN: 1613-978X

ARTICLE INFORMATION
==============================
PMCID: PMC9017731
DOI: 10.1007/s10273-022-3148-x
Article ID: 3148
Article version: 1
Subjects: Kommentare

Arzneimittel: Placebo bei Engpassbekämpfung

Pharmaceuticals: Placebo in combating bottlenecks

Blankart Katharina katharina.blankart@uni-due.de
1
Felder Stefan stefan.felder@unibas.ch
2
1 grid.5718.b 0000 0001 2187 5445 Universität Duisburg-Essen, Campus Essen Weststadttürme, Berliner Platz 6-8, 45127 Essen, Deutschland
2 grid.6612.3 0000 0004 1937 0642 Universität Basel, Peter Merian-Weg 6, 4002 Basel, Schweiz
Electronic publication date: 2022 Apr 19
Print publication date: 2022
Volume: 102
Issue: 4
First page: 248
Last page: 248
Copyright: © Der/die Autor:in 2022
License: Open Access: Dieser Artikel wird unter der Creative Commons Namensnennung 4.0 International Lizenz veröffentlicht (https://creativecommons.org/licenses/by/4.0/deed.de).
Open Access wird durch die ZBW — Leibniz-Informationszentrum Wirtschaft gefördert.
License URL: https://creativecommons.org/licenses/by/4.0/

Keywords: I18, D47
issue-copyright-statement: © The Author(s) 2022

==============================

Die neue Regierung hat sich im Koalitionsvertrag zum Ziel gesetzt, Lieferengpässe bei Medikamenten zu bekämpfen und geeignete Maßnahmen zu ergreifen. Dabei gibt es zwei Hebel: Kapazitäten und Preise. Bezüglich der Kapazitäten sollen die Maßnahmen die Herstellung bestimmter Arzneimittel teilweise zurückholen, um die Kapazitäten versorgungskritischer Wirkstoffe zu erhöhen. Der Abbau von Bürokratie und finanzielle Zuschüsse an Hersteller sollen für Versorgungssicherheit sorgen – in einem Markt mit mehreren tausend Wirkstoffen und hohen Rückverlagerungskosten eine gewaltige Aufgabe. Die Gesundheitspolitik befindet sich zunächst im Blindflug, betroffene Bereiche zu identifizieren. Zwar können die Hersteller seit 2018 Unterbrechungen ausgewählter Wirkstoffe an das Bundesinstitut für Arzneimittel und Medizinprodukte melden, doch es fehlen hinreichende Anreize dafür. Oft ist nicht bekannt, wo die Versorgung mit Arzneimitteln entlang der Lieferkette gefährdet ist.

Um Versorgungssicherheit zu gewährleisten, wären für bestimmte Wirkstoffe Aufschläge nötig. Der Theorie des Marktdesigns folgend sollten die Preise für die Vorhaltung von Produktionskapazität und das gelieferte Medikament getrennt betrachtet werden (Ockenfels, 2021). In Deutschland ist Versorgungssicherheit bisher kein Parameter bei der Preisfindung. Jedoch ist ähnlich wie bei Covid-19-Impfstoffen zu vermuten, dass der gesellschaftliche Wert ausgewählter Arzneimittel den gezahlten Preis übersteigt und Aufschläge für eine Vorhaltung rechtfertigt. Die Schweiz ist im Management von Engpässen einen Schritt weiter. Dort wurden lebenswichtige Medikamente definiert, für die Hersteller Engpässe an das zuständige Bundesamt für wirtschaftliche Landesversorgung melden. Zudem werden für kritische Bereiche Pflichtlager angelegt, wobei deren Effektivität unklar ist. Die auf privater Initiative basierende Datenbank drugshortage.ch erfasst darüber hinaus Engpässe im gesamten Arzneimittelmarkt. Kritiker beider Länder führen an, dass bei älteren Wirkstoffen mit abgelaufenem Patentschutz die Hersteller aufgrund niedriger Preise zu geringe Anreize hätten, ihre Produkte im Markt anzubieten und eine Vorhaltung zu garantieren. Die zusammengeführten Daten zu Engpässen von Arzneimitteln sowie zu Preisen aller in der Schweiz verfügbaren Präparate (Blankart and Felder, 2022) zeigen, dass etwa die Hälfte des verschreibungspflichtigen Marktes zwischen 2015 und 2020 von Verknappungen oder gar Ausfällen des Angebots betroffen war. Zeiträume, in denen vorübergehend kein Zugang zu allen infrage kommenden Arzneimitteln besteht (Versorgungsengpass), dauern im Median acht Monate und sind wesentlich länger, als wenn der Zugang nur teilweise eingeschränkt ist (Lieferengpass). Keine Unterschiede bestehen im Vergleich zur Situation bei lebenswichtigen Arzneimitteln, für die eine Meldepflicht gilt.

Während des ersten Lockdowns der COVID-19-Pandemie ist in der Schweiz die Rate der Engpässe für lebenswichtige Arzneimittel zurückgegangen, mutmaßlich parallel zum Rückgang der Nachfrage nach ambulanten und stationären Leistungen. Dagegen gab es keinen Anstieg von Engpässen bei den COVID-19-Behandlungen. Die Studie bietet auch Aufschluss über die Wirkung von Engpässen auf die Arzneimittelausgaben. Unter der Annahme, dass die verschriebenen Mengen insgesamt konstant bleiben, betrugen die zusätzlichen Ausgaben für 2018 zwischen 7 und 17 Mio. CHF (ca. 6,5 bis 16 Mio. Euro), sofern alle knapp gemeldeten Medikamente ausgetauscht wurden. Im Vergleich zu den Gesamtausgaben für Arzneimittel von 7,6 Mrd. CHF ein sehr kleiner Betrag. Interessanter ist die Beobachtung, dass bei der Hälfte der Engpässe eine im Markt teurere gleichwertige Alternative verfügbar war. Bei der anderen Hälfte waren die verfügbaren Alternativen günstiger als die knapp gemeldeten Arzneimittel. Es sind somit nicht ausschließlich die günstigsten Alternativen von Engpässen betroffen, und die zeitweise Knappheit führte in diesen Fällen sogar zu Ausgabensenkungen. Somit reicht es nicht, einzig auf Präparate mit geringen Preisen abzuzielen und Preise zu garantieren. Vielmehr sollten die Marktstruktur, der Umfang des Patentschutzes und die Zahl der Produktionsstandorte bekannt sein, um das Versorgungsrisiko abschätzen und eine angemessene Abgeltung von Vorhaltekosten bestimmen zu können.

Die Hersteller teurerer Arzneimittel unternehmen bereits Anstrengungen, um die Resilienz ihrer Lieferkette zu gewährleisten. In Märkten mit niedrigen Preisen mag es daran fehlen. Für die deutsche Gesundheitspolitik ist es daher geboten, vor der Ergreifung von Maßnahmen genauer zu prüfen, bei welchen Wirkstoffen keine genügende Versorgungssicherheit gegeben ist. Die internationale Arbeitsteilung in der Produktion von Arzneimitteln zurückzufahren kostet, und die Mittel zum Aufbau von lokalen Produktionskapazitäten sind begrenzt.


Literatur

Blankart, K. E. und Felder, S. (2022), Do Medicine Shortages Reduce Access and Increase Pharmaceutical Expenditure? A Retrospective Analysis of Switzerland 2015–2020, Value in Health [Preprint].10.1016/j.jval.2021.12.017 35219600
Ockenfels A Marktdesign für eine resiliente Impfstoffproduktion Perspektiven der Wirtschaftspolitik 2021 22 3 259 269 10.1515/pwp-2021-0031
